# Overall survival in advanced hepatocellular carcinoma treated with concomitant systemic therapy and stereotactic body radiation therapy or systemic therapy alone

**DOI:** 10.3389/fonc.2023.1290691

**Published:** 2023-11-27

**Authors:** Alexander Piening, Anand Swaminath, John Dombrowski, Ryan M. Teague, Noor Al-Hammadi, Jeevin Shahi

**Affiliations:** ^1^ Department of Molecular Microbiology and Immunology, Saint Louis University School of Medicine, St. Louis, MO, United States; ^2^ Department of Radiation Oncology, McMaster University, Hamilton, ON, Canada; ^3^ Department of Radiation Oncology, Saint Louis University School of Medicine, St. Louis, MO, United States; ^4^ Department of Health and Clinical Outcomes Research, Advanced HEAlth Data (AHEAD) Research Institute, Saint Louis University School of Medicine, St. Louis, MO, United States

**Keywords:** systemic therapy, stereotactic body radiation therapy, stereotactic radiosurgery, hepatocellular carcinoma, survival outcomes, retrospective analysis

## Abstract

**Introduction:**

First-line systemic therapy (ST) options for advanced hepatocellular carcinoma (HCC) include tyrosine kinase inhibitors and immunotherapy (IO). Evolving data suggest prolonged overall survival (OS) when ST is combined with stereotactic body radiation therapy (SBRT), although evidence is significantly limited in HCC populations. We hypothesized that advanced HCC patients in the National Cancer Database (NCDB) would have improved OS when receiving ST+SBRT vs ST alone.

**Methods:**

Stage III/IV HCC patients diagnosed from 2010-2020 and treated with first-line ST±SBRT were identified from the NCDB. The primary endpoint was OS from date of diagnosis stratified by the receipt of SBRT (ST+SBRT vs ST alone). Survival was estimated using Kaplan-Meier methodology and compared via log-rank. Multivariate analysis (MVA) was performed by Cox regression.

**Results:**

Of 10,505 eligible patients with stage III disease, 115 (1.1%) received ST+SBRT and 10,390 (98.9%) received ST alone. Of 9,617 eligible patients with stage IV disease, 127 (1.3%) received ST+SBRT and 9,490 (98.7%) received ST alone. Median follow-up time was 6.8 months. Baseline characteristics were similar between cohorts. Patients with stage III disease receiving ST+SBRT had improved median OS (12.62 months vs 8.38 months) and higher rates of survival at 1-year (53.0% vs 38.7%) and 2-years (27.0% vs 20.7%) compared to those receiving ST alone (log-rank *P*=0.0054). Similarly, patients with stage IV disease receiving ST+SBRT had improved median OS (11.79 months vs 5.72 months) and higher rates of survival at 1-year (49.6% vs 26.2%) and 2-years (23.6% vs 12.0%) (log-rank *P*<0.0001). On MVA, receipt of SBRT predicted improved OS (HR=0.748, 95%CI 0.588-0.951; *P*=0.0178) and receipt of IO trended towards improved OS (HR=0.859, 95%CI 0.735-1.003; *P*=0.0538).

**Conclusion:**

In advanced HCC, patients receiving ST+SBRT had improved OS compared to those receiving ST alone. Prospective clinical trials are warranted to better identify HCC populations which may benefit from combined modality therapy.

## Introduction

Hepatocellular carcinoma (HCC) is the third leading cause of cancer-related mortality worldwide and its incidence is expected to rise in the upcoming decades (1, 2). Curative-intent approaches for HCC include liver resection or transplantation, however up to 80% of patients are ineligible for these surgical treatments due to advanced stage at presentation, underlying liver dysfunction, medical comorbidities, poor performance status, and/or delays in liver transplantation (3). In non-surgical candidates, definitive locoregional therapy (LRT) options include radiofrequency ablation (RFA), percutaneous cryoablation, transarterial chemoembolization (TACE), transarterial radioembolization (TARE), and radiation therapy (RT) (4). More recently, there has been growing interest in stereotactic body radiation therapy (SBRT) as an LRT option for HCC. SBRT is a non-invasive technique allowing for the delivery of highly conformal and ablative radiation doses in five or fewer fractions. Unlike historical RT techniques for HCC, SBRT minimizes radiation doses to the uninvolved liver and surrounding tissues, significantly reducing the risk for radiation-induced liver disease (RILD) and other toxicities (5). Larger radiation doses per fraction with SBRT may improve local control (LC) compared to conventional RT, as the technique allows for a higher biologically effective dose (BED) to be delivered, and may instigate novel mechanisms of tumor cell killing such as vascular injury, necroptosis, and immunomodulation (6, 7). Clinical outcomes of SBRT for localized HCC are favorable, with long-term LC rates ranging from 80-90% (8, 9). As such, SBRT (either definitively or as a bridge to curative-intent surgery) is considered a standard LRT option by several evidence-based HCC treatment guidelines (4, 10, 11).

In advanced (stage III/IV) unresectable HCC, however, there is a lack of consensus on the role and feasibility of LRT. Patients with advanced HCC are often treated with systemic therapy (ST) alone due to macrovascular involvement, multifocal disease, or the presence of extrahepatic metastases; however, SBRT is feasible in these populations if liver function is maintained. For example, in RTOG 1112, Child-Pugh A patients with advanced HCC who were ineligible for surgery, ablation, and/or TACE were randomized to sorafenib with or without SBRT (12). Progression-free survival (PFS) and overall survival (OS) were prolonged in the cohort receiving combined sorafenib and SBRT, and no increased risk for toxicity was observed (12). These favorable results, along with recent randomized evidence demonstrating improved survival and safety of combined ST+SBRT for metastatic cancers, has led to significant interest in combining ST+SBRT for locally advanced and metastatic HCC (13–17). Combinatorial approaches have especially garnered interest in HCC patients receiving immunotherapy (IO), as the immunomodulatory effects of SBRT may synergistically enhance responses to IO (9, 18, 19). Although first-line IO options are now standard for advanced HCC, initial studies supporting liver SBRT in these populations were performed in the pre-IO era (4, 20, 21). It is unclear, therefore, whether the combination of SBRT with ST approaches incorporating IO would have similar benefits or potentially increase the risk of liver and/or other toxicities. Furthermore, stage IV HCC patients who have received ST+SBRT to the liver and/or extrahepatic sites are underrepresented in prior clinical trials on this topic, and it is unknown if this treatment strategy would improve survival endpoints in a contemporary population. Given the paucity of data evaluating combined ST+SBRT in HCC patients, we conducted a retrospective review of the National Cancer Database (NCDB) to analyze survival outcomes in stage III and stage IV HCC patients receiving first-line ST alone or ST+SBRT. We hypothesized that advanced HCC patients receiving ST+SBRT would have prolonged OS compared to those receiving ST alone.

## Materials and methods

### Data source

The NCDB was analyzed to identify patients diagnosed with HCC from 2010-2020. Data were obtained from the 2020 NCDB Participant User File. The NCDB is a joint project of the Commission on Cancer (CoC) of the American College of Surgeons and the American Cancer Society. The CoC’s NCDB and the hospitals participating in the CoC’s NCDB are the source of the de-identified data used herein; they have not verified and are not responsible for the statistical validity of the data analysis or the conclusions derived by the authors.

### Patient cohort

**Figure 1:**
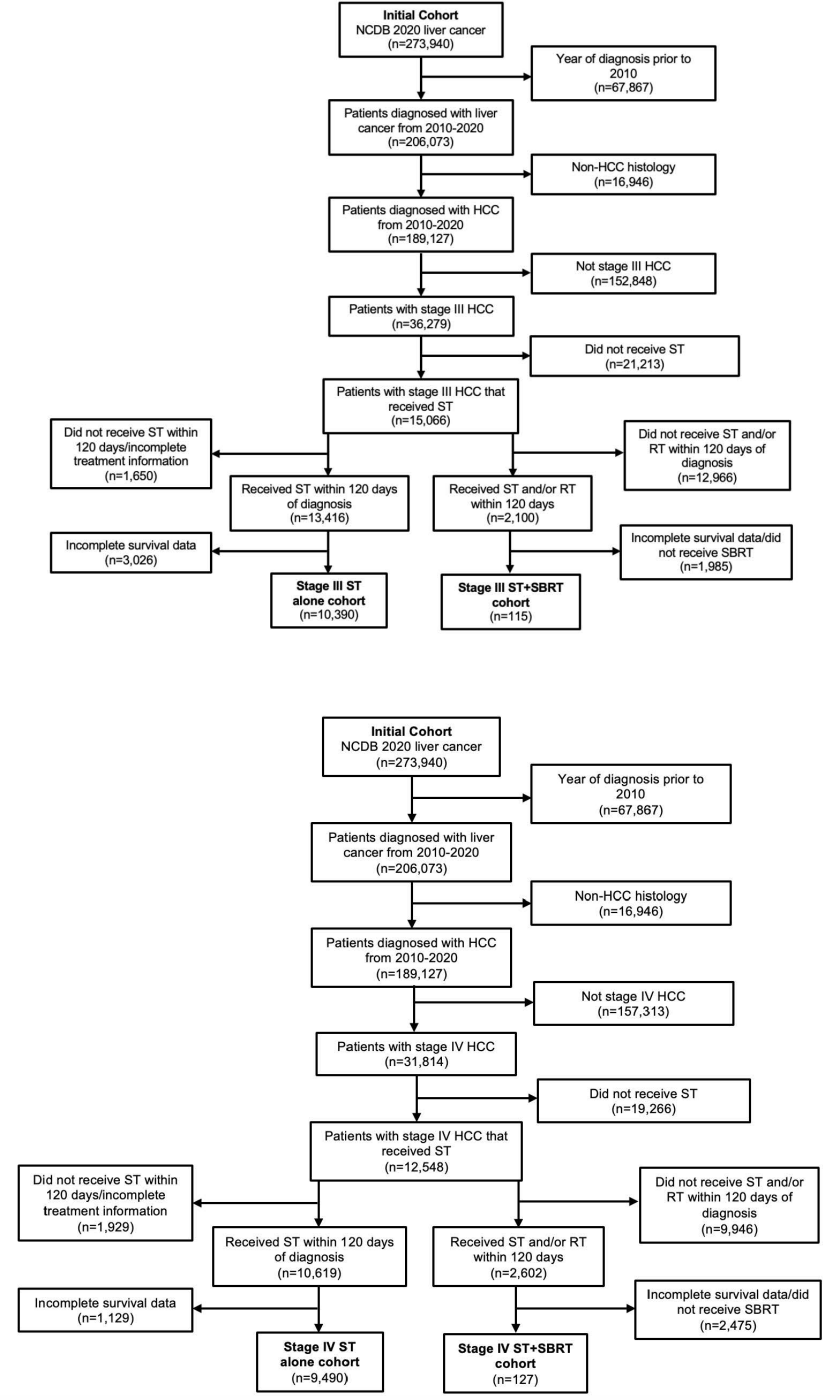
Consolidated Standards of Reporting Trials (CONSORT) diagram describing the selection of patients diagnosed with American Joint Committee on Cancer (AJCC) stage III and stage IV hepatocellular carcinoma (HCC) from the National Cancer Database (NCDB). Patients were included if they were treated with systemic therapy (ST) alone or combined ST and stereotactic body radiation therapy (SBRT).

Patients with American Joint Committee on Cancer (AJCC) stage III or stage IV HCC diagnosed from 2010-2020 and receiving ST with or without SBRT were identified from the NCDB. Eligible patients were required to have started treatment within 120 days of diagnosis and ST+SBRT patients were required to start secondary treatment (either ST or SBRT) within 120 days of the first treatment modality. Stage III patients received liver directed SBRT only, while stage IV patients received SBRT to the liver and/or extrahepatic sites. Patients with stage IV disease that received stereotactic radiosurgery to the brain or spine were included in the “SBRT” cohort. Complete AJCC staging details and radiation treatment characteristics were required for study inclusion. The dose/fractionation definition of SBRT was as follows: ≥10 Gy in 1 fraction, ≥20 Gy in 2 fractions, ≥24 Gy in 3 fractions, or ≥25 Gy in 5 fractions. Patients who underwent conventional (non-SBRT) radiation therapy courses (stage III n=199, stage IV n=1,340) were included in the ST alone cohort. Patients with incomplete data regarding demographics, therapy, AJCC tumor stage, and/or follow up time were excluded. Patient characteristics collected included age at diagnosis, sex, race/ethnicity, Charlson-Deyo score, median income, and facility type. Disease and treatment characteristics included primary tumor size, tumor grade, number of organ systems involved by metastasis, and details of radiation dose/fractionation. Screening criteria for study inclusion are outlined in **Figure 1**.

### Outcomes and statistical analysis

The primary endpoint was OS from date of diagnosis stratified by receipt of SBRT (ST+SBRT vs ST alone). Survival time was calculated from date of stage III or stage IV diagnosis to last contact or death. Events that occurred beyond 24 months were censored due to the limited expected follow-up time and low number of events to be captured beyond this time point. Baseline characteristics were compared using chi-squared tests for categorical variables based on treatment cohort. Summary statistics were provided for dose/fractionation of radiation therapy. Survival from date of diagnosis to death or last contact was estimated using Kaplan-Meier methodology and compared via the log-rank test. Univariate and multivariate (MVA) analysis was performed, and the hazard ratio (HR) estimated via Cox proportional hazards modeling. Analyses were performed using GraphPad Prism 9.0 and SAS statistical software (version 9.4; SAS Institute Inc, Cary, NC). This NCDB study was exempt from the local institutional review board.

## Results

Of 10,505 eligible patients with stage III disease, 115 (1.1%) received ST+SBRT and 10,390 (98.9%) received ST alone. Of 9,617 eligible patients with stage IV disease, 127 (1.3%) received ST+SBRT and 9,490 (98.7%) received ST alone. Stage III and stage IV patient characteristics are described in **Tables 1A**, **B**, respectively. Stage III and stage IV SBRT treatment characteristics are described in **Tables 2A**, **B**, respectively. Baseline characteristics were similar for stage III cohorts, although patients in the ST+SBRT cohort were more frequently treated at an academic/research program compared to the ST alone group (73.2% vs 53.2%, respectively, *P*=0.0002). Charlson-Deyo scores were similar between stage III ST alone and ST+SBRT cohorts (*P*=0.6407) with similar percentages of patients having a Charlson-Deyo score of 3 (16.8% vs 17.4%, respectively). For stage III patients, the ST alone cohort tended to have larger primary tumor sizes compared the ST+SBRT cohort (*P*=0.0451). In terms of systemic treatment types, the ST+SBRT cohort was more frequently treated with IO, or a combination of IO and “chemotherapy,” compared to the ST alone cohort (*P*=0.0158). Of note, the NCDB coding classifies tyrosine kinase inhibitors (TKIs) as “chemotherapy,” and the database does not offer additional granularity as to which specific ST agents were received by patients. The median time to treatment initiation was 37 days (range 1-120) for the stage III ST alone cohort and 67 days (range 1-119) for stage III ST+SBRT cohort. In the stage III ST alone cohort, 199 patients received conventional (non-SBRT) radiation therapy to the liver. For the 115 patients in the stage III ST+SBRT cohort, the median SBRT dose was 37.5 Gy (range 24-60), and the median number of fractions was 5 (range 3-5).

**Table 1A:** Patient characteristics for the stage III cohort receiving systemic therapy (ST) alone or combined ST and stereotactic body radiation therapy (SBRT).

Treatment	ST Alone (n=10390, 98.9%)	ST+SBRT (n=115, 1.1%)	P-value
Age at diagnosis			0.8760
<50	525 (5.1)	5 (4.3)	
50-59	2914 (28.0)	29 (25.2)	
60-69	4071 (39.2)	47 (40.9)	
70-79	2107 (20.3)	23 (20.0)	
>80	773 (7.4)	11 (9.6)	
Sex			0.0830
Male	8264 (79.5)	99 (86.1)	
Female	2126 (20.5)	16 (13.9)	
Race			0.7414
White	7374 (71.0)	85 (73.9)	
Black	1811 (17.4)	19 (16.5)	
Other	1205 (11.6)	11 (9.6)	
Charlson-Deyo Score			0.6407
0	5216 (50.2)	63 (54.8)	
1	2424 (23.3)	24 (20.9)	
2	1008 (9.7)	8 (7.0)	
3	1742 (16.8)	20 (17.4)	
Income			0.6019
0-25th percentile	2129 (23.8)	19 (19.6)	
26th-median	2176 (24.3)	21 (21.6)	
51st-75th percentile	2305 (25.7)	28 (28.9)	
>75th percentile	2343 (26.2)	29 (29.9)	
Facility type			*0.0002*
Community Cancer Program	425 (4.1)	2 (1.8)	
Comprehensive Community Cancer Program	2704 (26.4)	13 (11.6)	
Academic/Research Program	5456 (53.2)	82 (73.2)	
Integrated Network Cancer Program	1662 (16.2)	15 (13.4)	
Primary tumor size (mm)			*0.0451*
0-40	642 (11.1)	10 (19.6)	
41-70	2135 (37.0)	24 (47.1)	
71-100	1536 (26.6)	8 (15.7)	
>100	1454 (25.2)	9 (17.6)	
ST treatment type			*0.0158*
Chemotherapy*	9720 (93.6)	102 (87.9)	
Immunotherapy	388 (3.7)	6 (5.2)	
Both	282 (2.7)	8 (6.9)	
Median time to treatment initiation, days (range)	37 (1-120)	67 (1-119)	
N receiving non-SBRT radiation treatment to the liver	199 (1.9)	–	

*NCDB coding classifies tyrosine kinase inhibitors as chemotherapy.

Italic values represent statistical significance of *P*<0.05.

**Table 1B:** Patient characteristics for the stage IV cohort receiving systemic therapy (ST) alone or combined ST and stereotactic body radiation therapy (SBRT).

Treatment	ST Alone (n=9490, 98.7%)	ST+SBRT (n=127, 1.3%)	P-value
Age at diagnosis			0.1493
<50	638 (6.8)	11 (8.7)	
50-59	2698 (28.7)	32 (25.2)	
60-69	3754 (40.0)	51 (40.2)	
70-79	1835 (19.5)	21 (16.5)	
>80	467 (4.9)	12 (9.4)	
Sex			0.8436
Male	7706 (81.2)	104 (81.9)	
Female	1784 (18.8)	23 (18.1)	
Race			0.2310
White	6877 (72.5)	94 (74.0)	
Black	1719 (18.1)	17 (13.4)	
Other	894 (9.4)	16 (12.6)	
Charlson-Deyo Score			0.7736
0	5055 (53.3)	68 (53.5)	
1	2230 (23.5)	30 (23.6)	
2	925 (9.7)	15 (11.8)	
3	1280 (13.5)	14 (11.0)	
Income			0.2357
0-25th percentile	1945 (23.7)	22 (21.0)	
26th-median	1986 (24.2)	24 (22.9)	
51st-75th percentile	2173 (26.5)	23 (21.9)	
>75th percentile	2103 (25.6)	36 (34.3)	
Facility type			*<0.0001*
Community Cancer Program	560 (6.0)	6 (4.8)	
Comprehensive Community Cancer Program	2991 (32.2)	23 (18.5)	
Academic/Research Program	4125 (44.4)	81 (65.3)	
Integrated Network Cancer Program	1609 (17.3)	14 (11.3)	
Primary tumor size (mm)			0.5149
0-40	919 (20.4)	6 (16.2)	
41-70	1214 (26.9)	14 (37.8)	
71-100	1043 (23.1)	7 (18.9)	
>100	1330 (29.5)	10 (27.0)	
Brain metastasis at diagnosis			*<0.0001*
No	8781 (99.0)	111 (89.5)	
Yes	87 (1.0)	13 (10.5)	
Bone metastasis at diagnosis			*<0.0001*
No	6820 (76.7)	52 (41.6)	
Yes	2076 (23.3)	73 (58.4)	
Lung metastasis at diagnosis			*0.0057*
No	6526 (73.7)	105 (84.7)	
Yes	2331 (26.3)	19 (15.3)	
Distant lymph node metastasis at diagnosis			0.1654
No	3477 (83.6)	81 (89.0)	
Yes	683 (16.4)	10 (11.0)	
Metastasis at other site(s) at diagnosis			0.1330
No	3259 (78.3)	78 (84.8)	
Yes	905 (21.7)	14 (15.2)	
Organ systems involved by metastatic disease at diagnosis			0.3165
1	3821 (77.8)	74 (76.3)	
2	884 (18.0)	16 (16.5)	
3	171 (3.5)	5 (5.2)	
≥4	33 (0.7)	2 (2.1)	
ST treatment type			*0.0001*
Chemotherapy*	8695 (91.5)	103 (81.1)	
Immunotherapy	453 (4.8)	15 (11.8)	
Both	352 (3.7)	9 (8.1)	
Median time to treatment initiation, days (range)	34 (1-120)	45.5 (3-120)	
N receiving non-SBRT radiation treatment to any site	1340 (14.1)	-	

*NCDB coding classifies tyrosine kinase inhibitors as chemotherapy.

Italic values represent statistical significance of *P*<0.05.

**Table 2A:** Stereotactic body radiation therapy (SBRT) treatment characteristics for the stage III cohort receiving combined systemic therapy (ST) and liver SBRT.

Site treated (n=115)	Median dose (range)	Median number of fractions (range)
Liver	37.5 Gy (24-60)	5 (3-5)

Gy, Gray.

**Table 2B:** Stereotactic body radiation therapy (SBRT) treatment characteristics for the stage IV cohort receiving combined systemic therapy (ST) and SBRT.

Site treated (n=127)	Median dose (range)	Median number of fractions (range)
Spine (n=41, 32.3%)	24 Gy (12-40)	3 (1-5)
Liver (n=31, 24.4%)	35 Gy (14-60)	5 (1-5)
Non-spine bone (n=21, 16.5%)	25 Gy (20-50)	5 (1-5)
Other* (n=15, 11.8%)	30 Gy (14-40)	5 (1-5)
Brain (n=12, 9.4%)	24.5 Gy (18-30)	4 (1-5)
Chest/Lung (n=7, 5.5%)	40 Gy (24-51)	5 (3-5)

Gy, Gray.

*Includes abdominal lymph nodes, eye/orbit, head and neck, abdomen, and unspecified sites.

Baseline characteristics were also similar for stage IV cohorts, although once again patients in the ST+SBRT cohort were more likely to have received treatment at an academic/research program compared to the ST alone cohort (65.3% vs 44.4%, *P*<0.0001). Charlson-Deyo scores were similar between stage IV ST alone and ST+SBRT cohorts (*P*=0.7736) with similar percentages of patients having a Charlson-Deyo score of 3 (13.5% vs 11.0%). There was no difference in primary tumor size between the stage IV ST alone and ST+SBRT cohorts (*P*=0.5149). Compared to the ST alone cohort, the stage IV ST+SBRT cohort had higher rates of brain metastasis at diagnosis (10.5% vs 1.0%, *P*<0.0001) and bone metastasis at diagnosis (58.4% vs 23.3%, *P*<0.0001). In contrast, the ST alone cohort had higher rates of lung metastasis at diagnosis compared to the ST+SBRT cohort (26.3% vs 15.3%, *P*=0.0057). There was no significant difference in the number of organ systems involved by metastatic disease at diagnosis between the ST alone and ST+SBRT cohorts with stage IV disease (*P*=0.3165). Once again, in terms of systemic treatment types, the ST+SBRT cohort was more frequently treated with IO, or a combination of IO and “chemotherapy,” compared to the ST alone cohort (*P*=0.0001). The median time to treatment initiation was 34 days (range 1-120) for the stage IV ST alone cohort and 45.5 days (range 3-120) for the stage IV ST+SBRT cohort. In the stage IV ST alone cohort, 1,340 patients received conventional (non-SBRT) radiation therapy to any site. For the 127 patients in the stage IV ST+SBRT cohort, sites treated with SBRT included the spine (n=41, 32.3%), liver (n=31, 24.4%), non-spine bone (n=21, 16.5%), other sites (n=15, 11.8%), brain (n=12, 9.4%), and chest/lung (n=7, 5.5%).

**Figure 2:**
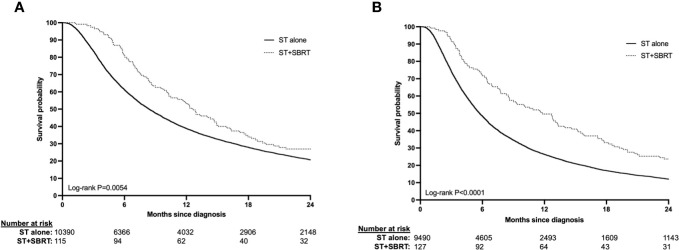
**(A, B)** Overall survival in stage III (2A) and stage IV (2B) HCC patients treated with systemic therapy (ST) alone or ST and stereotactic body radiation therapy (SBRT).

Patients with stage III disease receiving ST+SBRT had improved median OS (12.62 months vs. 8.38, log-rank *P*=0.0054) and higher rates of survival at 1-year (53.0% vs. 38.7%) and 2-years (27.0% vs. 20.7%) compared to those receiving ST alone (**Figure 2A**). Similarly, patients with stage IV disease receiving ST+SBRT had improved median OS (11.79 months vs 5.72 months, log-rank *P*<0.0001) and higher rates of survival at 1-year (49.6% vs 26.2%) and 2-years (23.6% vs. 12.0%) compared to those receiving ST alone (**Figure 2B**). Univariate and MVA analyses were performed using Cox proportional hazard regression of combined ST+SBRT therapy, disease stage (Stage III vs Stage IV), Charlson-Deyo score (0 vs >0), receipt of IO, and tumor size (<50mm vs > 50mm) to identify predictors of survival. On MVA, receipt of SBRT predicted for improved OS (HR=0.748, 95%CI 0.588-0.951; *P*=0.0178) and receipt of IO demonstrated a trend towards improved OS, although this did not reach statistical significance (HR=0.859, 95%CI 0.735-1.003; *P*=0.0538). Poor prognostic factors included stage IV disease (HR=1.509), primary tumor size > 5 cm (HR=1.354), and a Charlson-Deyo Score >0 (HR=1.106) (all *P*<0.0001) (**Table 3**, **Supplementary Table 1**). Additional subgroup analyses were performed on stage III and stage IV ST+SBRT cohorts to investigate the influence of treatment modality sequencing (ST then SBRT vs SBRT then ST) on OS. Stratification of these groups based on treatment modality sequencing revealed no differences in OS for both stage III and stage IV cohorts (log-rank *P*=0.3615 and *P*=0.8843, respectively).

**Table 3:** Univariate and multivariate analyses.

	Univariate		Multivariate			
Predictors	HR	95% CI	P-value	HR	95% CI	P-value
Received ST+SBRT	0.737	0.580-0.937	*0.013*	0.748	0.588-0.951	*0.0178*
Stage IV Disease	1.426	1.367-1.489	*<0.0001*	1.509	1.444-1.576	*<0.0001*
Charlson-Deyo Score > 0	1.073	1.028-1.119	*0.0013*	1.106	1.059-1.154	*<0.0001*
Received Immunotherapy	0.905	0.775-1.056	0.2056	0.859	0.735-1.003	0.0538
Tumor size > 50mm	1.225	1.162-1.291	*<0.0001*	1.354	1.283-1.428	*<0.0001*

ST, systemic therapy; SBRT, stereotactic body radiation therapy; HR, hazard ratio; CI, confidence interval.

Italic values represent statistical significance of *P*<0.05.

## Discussion

In this large retrospective analysis of the NCDB, we found significantly improved OS in advanced (AJCC stage III/IV) HCC patients receiving first-line ST+SBRT compared to ST alone. In stage III patients, median survival (12.62 months vs 8.38 months), and rates of 1-year (53.0% vs 38.7%), and 2-year (27.0% vs 20.7%) survival were improved in patients receiving ST+SBRT vs ST alone, respectively (log-rank *P*=0.0054). Similarly in stage IV disease, median survival (11.79 months vs 5.72 months), as well as rates of 1-year (49.6% vs 26.2%), and 2-year (23.6% vs 12.0%) survival were superior in the ST+SBRT cohort (log-rank *P*<0.0001). On multivariate analysis of all patients, the receipt of SBRT was predictive for improved OS when adjusting for disease stage, performance status, receipt of IO, and tumor size (HR=0.748, 95%CI 0.588-0.951; *P*=0.0178). Negative prognostic factors for OS were stage IV disease (HR=1.509), primary tumor size > 5 cm (HR=1.354), and a Charlson-Deyo Score >0 (HR=1.106) (all *P*<0.0001). These results provide evidence for considering combined ST+SBRT in patients with advanced HCC and demonstrate the need for further prospective studies evaluating this treatment approach.

While TKIs and/or IO agents are first-line treatment options for advanced HCC, there is limited randomized data evaluating whether combining local treatment of gross disease with ST improves survival outcomes (4, 20–22). This strategy was tested in RTOG 1112, a randomized phase III trial of sorafenib with or without SBRT in Barcelona Clinic Liver Cancer (BCLC) stage B/C HCC patients (n=193) (12). The investigators noted a significant improvement in median OS from 12.3 months to 15.8 months with combined SBRT and sorafenib, with no increase in treatment-related toxicity. Nearly all (96%) of the advanced HCC patients in RTOG 1112 did not have metastatic disease and, therefore, the extrahepatic disease burden in these patients would be similar to the stage III HCC patients evaluated in this NCDB analysis. The more favorable median OS in RTOG 1112, however, may be explained by the fact that 40% of patients had a solitary lesion, whereas all AJCC stage III HCC patients in our study (by definition) had higher-risk disease with either multifocal tumors, vascular invasion, or locally advanced disease. Although it is difficult to compare survival endpoints between these different study populations, the median OS benefit of SBRT in RTOG 1112 (3.5 months) was similar to stage III HCC patients who received liver SBRT in this NCDB analysis (4.2 months). A limitation of the NCDB, however, is that it is not possible to abstract the specific ST regimen received by each individual patient. Because eligible patients in this NCDB analysis were treated in a similar time period as RTOG 1112 (2010–2020), it is expected that the majority of stage III patients received sorafenib as their first-line ST agent. This presumption is consistent with our study’s stage III patient characteristics, as 87.9% and 93.6% of ST+SBRT and ST alone patients, respectively, received “chemotherapy” alone (TKIs are classified as “chemotherapy” in the NCDB). Liver SBRT doses in our analysis (median: 37.5 Gy in 5 fractions) were also comparable to those in RTOG 1112 (range: 27.5-50 Gy in 5 fractions). While again it is difficult to directly compare the results of this retrospective NCDB analysis to RTOG 1112, the analogous outcomes in stage III disease strongly suggest a benefit to combined modality therapy in this patient population. Interestingly, the observed survival advantage with SBRT in these two studies is in contrast to three negative randomized trials of Yttrium-90 (Y90) TARE with or without sorafenib in localized HCC (23–25). As compared to Y90 and other LRT options for localized HCC, SBRT may have several potential advantages: (1) the incorporation of physician-defined margins for microscopic disease and geometric uncertainties in radiation delivery; (2) the ability to treat multiple hepatic and/or extrahepatic targets within a monoisocentric or separate radiation field; (3) enhanced tumoral responses and normal tissue repair secondary to fractionation of total radiation dose; (4) non-invasive treatment and lack of any significant anatomical limitations to radiation delivery (i.e., heat-sink effect with RFA, lung shunt fraction with Y90 TARE), and (5) possible abscopal and immunomodulatory responses to SBRT which may enhance systemic disease control (6). Given the lack of comparative and randomized evidence, however, it remains unclear which LRT option provides superior disease control. We strongly recommend multidisciplinary input in patients eligible for LRT, particularly when such approaches are combined with ST.

In stage IV disease, OS was also found to be significantly prolonged in the cohort receiving ST+SBRT, with a near-doubling of median OS (5.72 to 11.79 months) and the proportion of patients alive at 1- (26.2% vs 49.6%) and 2-years (12.0% to 23.6%) following diagnosis. There was no difference in metastatic disease burden in patients receiving ST alone or ST+SBRT, suggesting a balanced degree of extrahepatic disease between these two cohorts. Historically, ST alone was considered a standard approach in stage IV HCC; however, these results shed light on the idea that the ablation of locoregional and/or distant metastatic disease with SBRT may improve survival outcomes. In fact, several randomized trials in various cancer subtypes have demonstrated improved PFS and OS in stage IV patients undergoing ST with SBRT (13–17). The benefits of SBRT in this setting may also extend to patient quality of life, as SBRT to progressive sites may prevent complications and/or delay changes to potentially more toxic (and costly) next-line ST (26). This latter strategy may be particularly advantageous in HCC patients who have previously undergone a liver transplant and are receiving immunomodulatory drugs which may preclude them from receiving IO. In this setting, SBRT may offer these patients another “line” of therapy, whereby progressive sites can be treated with SBRT, and the patient can be maintained on their current ST strategy or observation. On the other hand, patients receiving IO may benefit from combined treatment with SBRT, as accumulating data reveal synergism when these treatment approaches are combined. For example, stereotactic radiation therapy may promote the expansion of tumor-antigen specific T cells and increase tumoral MHC-I expression, leading to more effective T cell-mediated killing (27, 28). Although IO is now a first-line option for advanced HCC, only 3.7% (ST alone) and 5.2% (ST+SBRT) of the stage III cohorts, and 4.8% (ST alone) and 11.8% (ST+SBRT) received IO in this analysis. This is a limitation of this study, as due to the period in which patient data was collected (2010-2020), it is likely that the majority of patients received first-line TKI therapy, and, thus, our results may not be generalizable to a more modern HCC population. For example, a patient captured from 2010 in our study is more likely to have received first-line TKI therapy than a patient captured in 2019 who would have likely been treated with an IO agent. To that end, it is unclear if the survival benefit of SBRT observed in this retrospective analysis would hold true if modern ST regimens incorporating IO were consistently utilized in our population. Immunotherapy use did demonstrate a trend towards improved OS on MVA, however, this was not statistically significant (HR=0.859, 95%CI 0.735-1.003; *P*=0.0538). In addition, the benefit of SBRT will need to be further assessed as next-generation sequencing techniques are utilized to develop personalized targeted drug therapies and new immunotherapy agents are introduced.

Although outside of the scope of this retrospective analysis, it is important to consider that there may be an increased risk for SBRT-related toxicities when radiation is delivered concurrently (or sequentially) with ST agents (29). While the majority of liver SBRT experiences report rates of grade 3 or greater toxicities to be less than 10%, this risk may be amplified in HCC patients with Child Pugh B/C liver dysfunction, extensive primary tumors, extrahepatic metastases in high-risk anatomic sites (such as the central lung/mediastinum), abutment of critical organs-at-risk (OARs), and in those receiving immunosuppressive medications (8, 9). Common abdominal OARs include the small/large bowel, duodenum, esophagus, and biliary tree; however, in stage IV disease, any anatomic site is potentially at risk. For example, the most common metastatic sites that received SBRT in our analysis were the spine, bones, brain, and chest/lungs; therefore, physician and institutional experience in providing multi-site SBRT is recommended when treating advanced HCC patients. This is particularly important when delivering SBRT in patients who have received antiangiogenic therapy (such as bevacizumab), as severe and grade 5 toxicities (most commonly fatal hemorrhage or bowel perforation) have been reported in the literature (30, 31). We look forward to the final results of ongoing phase I/II trials (ClinicalTrials.gov identifiers: NCT03817736, NCT03203304, NCT03316872) evaluating the role of combined SBRT and IO for advanced HCC, as this is a topic of growing interest and will offer further insights into the efficacy and potential toxicities of combined SBRT+IO.

There are several limitations to this study, chiefly its retrospective nature, which may introduce significant selection bias as well as inability to control for potentially confounding factors such as HCC etiology, Child-Pugh score, and treatment-related adverse events. Next, although data collected by the NCDB is done so at CoC-accredited hospitals, this data is often incomplete and can lead to errors in data analysis. We only included patients with complete AJCC staging and treatment information, which may limit the impact of any inconsistencies in data abstraction. The NCDB, however, does not adequately allow for the assessment of oligometastatic tumor burden (total number of metastases), which may bias our results toward improved outcomes in stage IV patients who received SBRT for a limited total number of metastases. We attempted to correct for this limitation by evaluating the metastatic tumor burden of patients, defined as the number of organ systems involved by metastatic disease at diagnosis. In the stage IV cohorts with evaluable data, there was no difference in metastatic tumor burden between these two groups. Other limitations include the small sample size of patients undergoing ST+SBRT in stage III (n=115, 1.1%) and stage IV populations (n=127, 1.3%), which may limit the power of some analyses, and lack of a known indication for SBRT. Patients in our analysis were diagnosed between 2010-2020 and may have received a variety of ST regimens. A significant limitation of the NCDB is that it does not contain specifics regarding which ST agents were provided to patients. Our results may, therefore, not be generalizable to modern patients who more commonly receive IO as part of their first-line treatment.

In this retrospective NCDB analysis of advanced (AJCC stage III/IV) HCC patients undergoing first-line ST with or without SBRT, it was found that those receiving ST+SBRT had improved median OS and rates of long-term survival at 1- and 2-years. On MVA, both stage III and stage IV patients significantly benefited from the addition of SBRT to ST. The results of this study demonstrate the need for further randomized prospective studies investigating the potential advantages and toxicities of combined modality therapy for advanced HCC. We propose that combined SBRT and modern doublet therapy ST strategies be considered in future prospective trials. Factors such as ST mechanism of action, SBRT dose/volume, immunotherapy use, oligometastatic disease burden, and liver transplant status should be considered in future analyses of HCC patients receiving ST+SBRT.

## Data avalability statement

The data analyzed in this study is subject to the following licenses/restrictions: Datasets analyzed in this study were made available to the researchers by request to the American College of Surgeons National Cancer Database. Requests to access these datasets should be directed to https://www.facs.org/quality-programs/cancer-programs/national-cancer-database/.

## Ethics statement

This National Cancer Database Analysis study was exempt from the local institutional review board.

## Author contributions

AP: Data curation, Formal Analysis, Investigation, Methodology, Visualization, Writing – original draft, Writing – review & editing. AS: Writing – review & editing. JD: Writing – review & editing. RT: Writing – review & editing. NA-H: Data curation, Formal Analysis, Software, Writing – review & editing. JS: Conceptualization, Data curation, Formal Analysis, Investigation, Project administration, Supervision, Writing – original draft, Writing – review & editing.
