# Supplementary material for: Overall survival in advanced hepatocellular carcinoma treated with concomitant systemic therapy and stereotactic body radiation therapy or systemic therapy alone
Source: Front Oncol. 2023 Nov 27;13:1290691. doi: 10.3389/fonc.2023.1290691 (PMC10711055; doi:10.3389/fonc.2023.1290691)
Supplement: Supplementary file 1 [file Table_1.docx]

**Supplementary Table 1.** Cox proportional hazards regression.

|  | **Model 1*** | | **Model 2 – Stage III** | | | | **Model 3 – Stage IV** | | |
| --- | --- | --- | --- | --- | --- | --- | --- | --- | --- |
| **Predictors** | **HR** | **95% CI** | **P-value** | **HR** | **95% CI** | **P-value** | **HR** | **95% CI** | **P-value** |
| Received ST+SBRT | 0.732 | 0.576-0.931 | *0.011* | 0.84 | 0.613-1.152 | 0.2804 | 0.637 | 0.439-0.924 | *0.0174* |
| Stage IV Disease | 1.427 | 1.367-1.489 | *<0.0001* |  |  |  |  |  |  |
| Charlson-Deyo Score > 0 |  |  |  | 1.102 | 1.072-1.137 | *0.019* | 1.148 | 1.078-1.222 | *<0.0001* |
| Received Immunotherapy |  |  |  | 0.941 | 0.744-1.190 | 0.611 | 0.802 | 0.653-0.985 | *0.0358* |
| Tumor size > 50mm |  |  |  | 1.063 | 1.063-1.246 | *0.0005* | 1.517 | 1.413-1.629 | *<0.0001* |

*Interaction term between stage and ST+SBRT was significant (tested but model did not converge).

Abbreviations: ST = systemic therapy; SBRT = stereotactic body radiation therapy; HR = hazard ratio, CI = confidence interval
